# Identification and functional comparison of novel alternatively spliced isoforms of human YAP

**DOI:** 10.1002/2211-5463.13618

**Published:** 2023-05-08

**Authors:** Lianlian Liu, Junlei Zhang, Jiaqi Wang, Yanping Tian, Jiali Wang, Yixiao Xu, Yuda Cheng, Meng Yu, Jiangjun Wang, Yi Yang, Xueyue Wang, Ran Yang, Wei Wu, Chen Zhang, Yan Hu, Rui Jian, Lan Xiao, Yan Ruan

**Affiliations:** ^1^ Laboratory of Stem Cell & Developmental Biology, Department of Histology and Embryology Army Medical University Chongqing China; ^2^ Department of Histology and Embryology, Chongqing Key Laboratory of Neurobiology, Brain and Intelligence Research Key Laboratory of Chongqing Education Commission Army Medical University Chongqing China; ^3^ Department of Pathophysiology, College of High Altitude Military Medicine Army Medical University Chongqing China; ^4^ Joint Surgery Center, Southwest Hospital Army Medical University Chongqing China; ^5^ Department of Cell Biology, College of Basic Medicine Army Medical University Chongqing China; ^6^ Experimental Center of Basic Medicine, College of Basic Medical Sciences Army Medical University Chongqing China; ^7^ Department of Pediatrics The General Hospital of PLA Tibet Military Area Command Lhasa China; ^8^ Thoracic Surgery Department Southwest Hospital, The First Hospital Affiliated to Army Medical University Chongqing China; ^9^ Department of Military Basic Training and Army Management, Army Health Service Training Base Army Medical University Chongqing China

**Keywords:** alternative splicing, cell chemosensitivity, transcriptional activation ability, YAP

## Abstract

As a key effector of the Hippo pathway, yes‐associated protein (YAP) is a major regulator of cell proliferation and apoptosis. In this study, 23 hYAP isoforms were identified in HEK293 cells, with 14 isoforms being reported for the first time. These isoforms were classified into hYAP‐a and hYAP‐b isoforms based on the variation in exon 1. The two groups of isoforms showed distinctly different subcellular localizations. hYAP‐a isoforms could activate TEAD‐ or P73‐mediated transcription, affect the proliferation rate, and enhance the cellular chemosensitivity of HEK293 cells. Moreover, different activation abilities and pro‐cytotoxic effects were observed among hYAP‐a isoforms. However, hYAP‐b isoforms were not found to exert any significant biological effects. Our findings add to the knowledge of YAP gene structure and protein‐coding capacity and will help in the elucidation of the function and related molecular mechanisms of the Hippo‐YAP signaling pathway.

## Graphical abstract

Fourteen novel isoforms of human YAP have been identified, bringing the total number of isoforms to 23. These isoforms were classified into hYAP‐a and hYAP‐b groups according to differences in exon 1. Functional comparison indicated that YAP‐a isoforms activate TEAD‐ or P73‐mediated transcription, affect proliferation rate, and enhance chemosensitivity. However, hYAP‐b isoforms were not found to exert any significant biological effects.

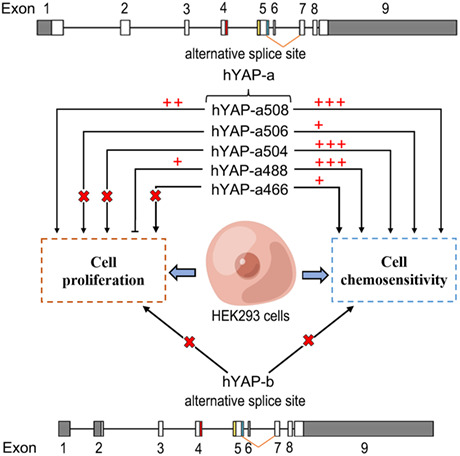

**Abbreviations**
AS: alternative splicing
FBS: fetal bovine serum
hP73α: human P73α
hYAP: human YAP
KD: knockdown
MW: molecular weights
OE: overexpression
ORFs: open reading frames
TAD: transcriptional activation domain
TSS: translation start site
YAP: yes‐associated protein

Alternative pre‐mRNA splicing is the process by which exons are assembled to form multiple mature mRNAs [1]. The major types of alternative splicing (AS) events include exon skipping, intron retention, alternative 3′ or 5′ splicing, and mutually exclusive exons [2]. As a crucial post‐transcriptional regulatory mechanism, AS helps generate protein isoforms with different structures and distinct or even antagonistic biological functions [3, 4]. It has been reported that around 95% of human genes undergo AS [5]. Moreover, aberrant alternative splicing of tumor suppressor genes and oncogenes might systematically affect an entire cancer‐associated process that drives the transformation of normal cells into malignant cells [6, 7]. Therefore, the exploration of isoforms not only is a prerequisite for the study of gene function but can also provide novel insights into the mechanisms underlying tumorigenesis.

The Hippo signaling pathway is an evolutionarily conserved network that plays a crucial role in maintaining organ size. The core components of the canonical Hippo pathway in mammals include MST1/2, SAV1, LATS1/2, MOB1A/B, and YAP/TAZ. YAP is the key effector of this pathway. When the Hippo pathway is inactive, YAP translocate to the nucleus, and forms a complex with the transcription factors to regulate the expression of their downstream target genes, thus affecting cell proliferation, apoptosis, and drug resistance [8, 9, 10].

The human *YAP* (*hYAP*) gene contains nine exons and encodes a 65‐kDa protein. hYAP protein comprises a TEAD factor‐binding domain, one or two WW domains, an SH3‐binding domain, a transcriptional activation domain (TAD), and a PDZ‐binding motif [11]. As a transcriptional coactivator, YAP can interact with various transcription factors. The TEAD family transcription factors have been identified as the major DNA‐binding partners of YAP [12, 13, 14]. Moreover, YAP can also bind to PPxY‐containing proteins, such as RUNX1/2 [15], ErbB‐4 [16, 17], TBX5 [18], and P73 [19, 20], through the WW domain, or to ZO‐2 through PDZ‐binding motif [14].

Yes‐associated protein is evolutionarily highly conserved. YAP isoforms have been identified from arthropods to vertebrates. We have previously identified eight different isoforms of mouse Yap. Each of these isoforms exhibited a different role in regulating self‐renewal maintenance, pluripotency exiting, and differentiation of mouse embryonic stem cells [21, 22]. According to NCBI gene annotations, there are nine isoforms of hYAP. Of these, eight isoforms (hYAP‐v1/v2/v3/v5/v6/v7/v8/v9) have the same transcription start sites (TSS). The ninth isoform (hYAP‐v4) transcribes from downstream TSS, producing proteins with truncated N terminus. The eight isoforms with intact N terminus can be divided into YAP1–1 and YAP1–2 groups based on the inclusion or exclusion of exon 4 (coding for the WW2 domain), and each group can be further divided into α, β, γ, and δ subtypes [23, 24]. Although some evidence supports their different transactivation abilities [24], it is still unclear whether more hYAP isoforms exist and, if so, how they exert their biological functions.

In this study, we discovered 14 novel alternative isoforms of hYAP. Furthermore, the differences in molecular (transactivation ability) and cellular (drug resistance) functions of the different hYAP isoforms were systematically analyzed. These findings not only lay the foundation for understanding the function and regulatory mechanism of the YAP gene but also help elucidate the role of the Hippo signaling pathway in related diseases.

## Materials and methods

### HEK293 cells culture and transfection

Cell lines including A549, H460, A375, A2058, A673, Saos2, U87, SW‐13, Hela, SW1990, MDA‐MB‐231, SW480, LNCaP, PC‐3, MRC5, and HEK293 were purchased from Cobioer Biosciences Co., Ltd. (Nanjing City, China) Cell lines (A549, H460, A375, A2058, A673, Saos2, U87, SW‐13, Hela, SW1990, MDA‐MB‐231, SW480, LNCaP, PC‐3, MRC5, and HEK293) were cultured in DMEM (Hyclone, South Logan, UT, USA), supplemented with 5% fetal bovine serum (FBS) (Gibco, South Logan, UT, USA), 2 mm GlutaMAX, and 0.1 mm non‐essential amino acids. hESC (Wicell, Madison, USA) cells were cultured in mTeSR™1 (Stem Cell Technologies). All cell cultures were maintained at 37 °C under 5% CO2. Transfection with Lipofectamine 2000 (Invitrogen, Carlsbad, California, USA) was performed according to the manufacturer's instructions. For stable transfection, the cells were cultured in the presence of 100 μg·mL^−1^ hygromycin B (Invitrogen). The resistant colonies were pooled and expanded for further analysis.

### Rapid amplification of cDNA ends (RACE)

5′‐RACE and 3′‐RACE were performed on the RNA extracted from HEK293 cells using a SMARTer RACE 5′/3′ kit, according to the manufacturer's instructions. Primers for the *hYAP* sequence were synthesized by Sangon Biotech Co. Ltd. (Shanghai, China). The prepared complementary DNA (cDNA) samples were subjected to PCR with 3′‐RACE primer 5′‐CCTCACAGCAGAACCGTTTCCCAGAC‐3′ or 5′‐RACE primer 5′‐AACCTGCTGGCAGAGGTACATCATCAG‐3′ and a universal primer mixture. The products were subjected to agarose gel electrophoresis, and the resolved fragments were purified. The purified fragments were then cloned into pGEM‐T Easy (Promega, Madison, Wisconsin, USA) and sequenced (BGI, Beijing Genomics Institution).

### Plasmid construction

Short hairpin RNA (shRNA) of human P73 targeting sequence (GACGAGGACACGTACTACC) was designed and subjected to BLAST analysis to ensure specificity. The shRNA against human P73 and a non‐target shRNA (GACGAACACTTCTTCATCG) were cloned into the lentiviral vector pLL3.7puro [25, 26]. The full‐length open reading frames (ORFs) of the *YAP* isoforms, human *P73α* (*hP73α*), and *TEAD2* were PCR amplified from the cDNA of the HEK293 cells using KOD‐Plus‐ (TOYOBO, Osaka, Japan) and cloned into pGEM‐T Easy (Promega). After DNA sequence verification, the ORFs were subcloned into pPyCAGIH or pPyCAGIZ [27]. The 3×FLAG and GFP fragments were PCR amplified and subcloned in‐frame into expression vectors. The P73‐responsive luciferase reporter construct was obtained by subcloning the PCR‐amplified fragment (−715 to −317 bp) from the BAX gene promoter into BglII‐HindIII sites of the pGL3‐luciferase Enhancer vector (Promega) [28]. HIP (negative control)/HOP (8 × WT TEAD‐binding site)‐flash reporter system was a gift from Barry Gumbiner (Addgene plasmid #83466, #83467) [29]. The primers used for plasmid construction are listed in Table S1. The DNA sequences of the plasmids are available upon request.

### Lentiviral production and infection

Lentiviral production and infection were performed as described previously [22].

### RNA isolation, reverse transcription (RT), semi‐quantitative RT‐PCR, and quantitative real‐time PCR

RNA isolation, RT, semi‐quantitative PCR, and quantitative real‐time PCR were performed as described [22]. Briefly, we used nested PCR to perform semi‐quantitative RT‐PCR. The sequences of the primers used for these PCRs are listed in Tables S2 and S3.

### Subcellular localization analysis

Subcellular localization was analyzed as described previously [22]. Fluorescent images were acquired using a Zeiss LSM780 NLO (Carl Zeiss Microscopy GmbH, Gottingen, Germany) confocal microscope.

### Luciferase assay

Luciferase reporter (0.4 μg) was co‐transfected into HEK293 cells with 0.01 μg pRL‐SV40 (Promega) as the internal control. The transfected cells were cultured for 48 h. To disrupt the YAP‐TEAD interaction, the cells were treated with 5 μm verteporfin (Selleck, Houston, TX, USA) for 48 h. The cells were then collected, and the luciferase assay was performed according to the instructions of the manufacturer of the Dual‐Luciferase Reporter System (Promega). The activity of the luciferase gene was normalized against that of Renilla.

### CCK‐8 assay

Cell proliferation assay was performed using the Cell Counting Kit‐8 (Beyotime, Shanghai, China), as per the manufacturer's instructions. Cells were seeded in 96‐well plates at a density of 1 × 10^4^ cells per well for 72 h and incubated with 10 μL CCK‐8 at 37 °C for 60 min in a culture comprising 5% fetal bovine serum (FBS) medium supplemented with either 50 μg·mL^−1^ cisplatin (Sigma, St. Louis, MO, USA) or 4 μm adriamycin (Selleckchem). The absorbance of the culture was then measured at 450 nm using an enzyme marker (Thermo Scientific Multiskan SkyHigh, Waltham, Massachusetts, USA).

### Co‐immunoprecipitation (Co‐IP) assay and western blot (WB) analysis

Co‐IP assay and WB analysis were performed as described previously [21]. The antibodies used in these procedures are listed in Table S4.

### Apoptosis assay

The apoptosis assay was performed as described previously [22], with minor modifications. Briefly, HEK293 cells were grown for 72 h in the presence of either 50 μg·mL^−1^ cisplatin or 4 μm adriamycin. The cultured cells were then harvested and stained with annexin V‐APC and PI. Flow cytometric analysis (NovoCyteTM D3130, ACEA Bioscience, San Diego, California, USA) was performed using an apoptosis detection kit (Biolegend, San Diego, California, USA) as per the manufacturer's instructions. Data were analyzed using the novoexpress software (ACEA Biosciences Inc, San Diego, California, USA). All measurements were done under the same instrument settings. Twenty thousand cells per sample were analyzed.

### Cell cycle analysis

After seeding HEK293 cells into 6‐well plates for 72 h, cell layers were digested with trypsin and fixed with 70% ethanol at −20 °C overnight. Subsequently, the cells were washed with phosphate‐buffered saline (PBS), and the cell precipitate was collected. Next, 100 μL of solution [containing 90 μL PBS, 10 μL Triton X‐100, 0.1 μL RNase A (0.2%), and 100 μg·mL^−1^, Sigma] was added into 1.5 mL EP tubes to resuspend the cells. The tubes were incubated at 37 °C for 30 min. Then, 100 μL of staining solution (containing 100 μL PBS, 1 μL PI, and 50 μg·mL^−1^, Sigma) was added to the EP tubes. The solution in the tubes was mixed well, incubated in the dark for 10 min, and analyzed using flow cytometry (NovoCyte D3130, ACEA Bioscience). Data were analyzed using the noveexpress software.

### Statistical analysis

The Statistical Package for Social Sciences (spss for Windows package release 25.0, Chicago, IL, USA) was used for statistical analysis of variance via Student's *t*‐test and Dunnett's *t*‐test. Data were plotted as mean ± SD, and *P* < 0.05 was considered statistically significant. Each experiment was conducted at least three times.

## Results

### Identification of hYAP isoforms

**Fig. 1:**
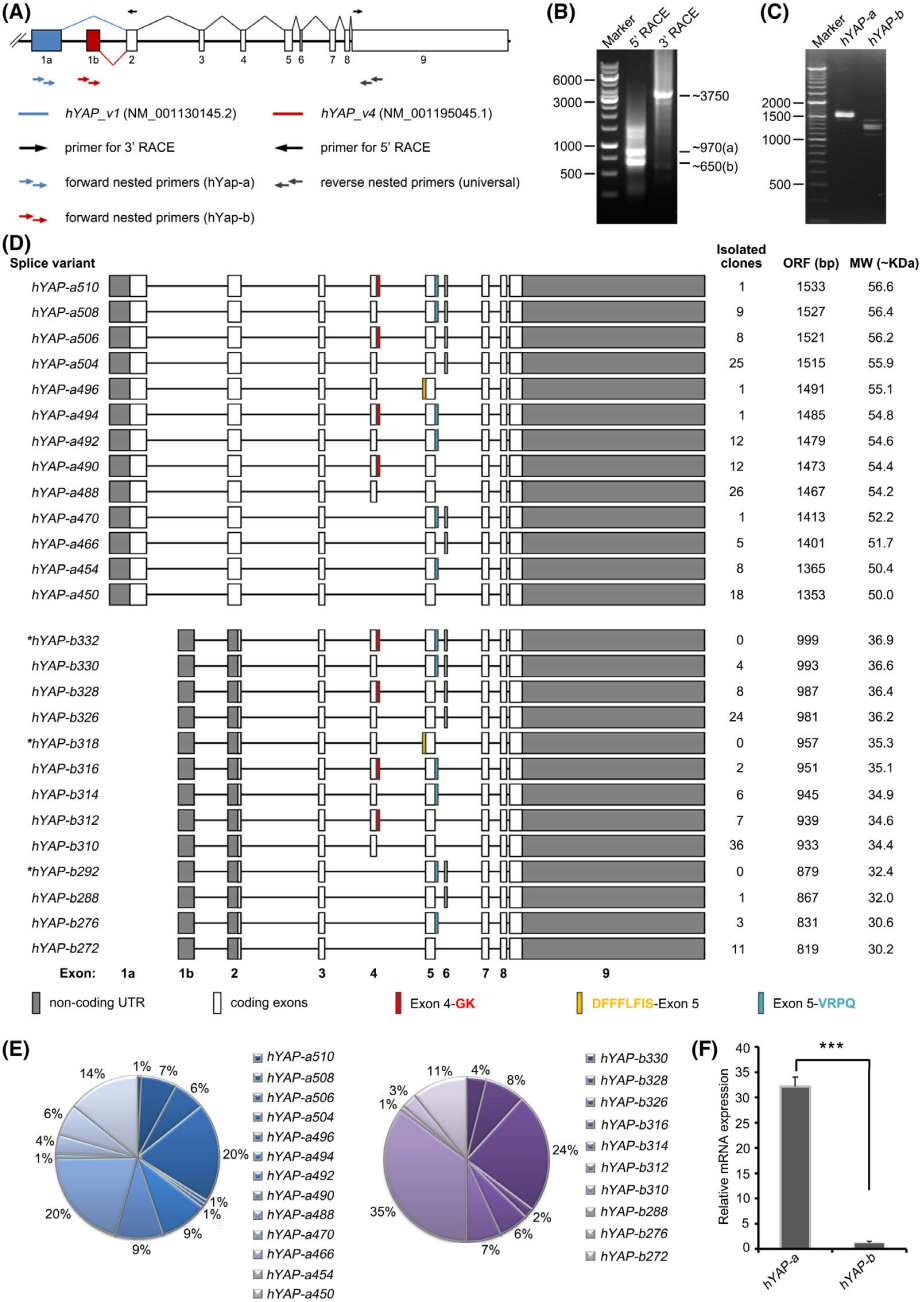
Identification of *hYAP* isoforms. (A) Gene structures of hYAP‐v1 and hYAP‐v4. All coding exons are indicated by numbered boxes. The positions of primers for RACE and nested PCR were indicated by arrows. (B) Amplification of transcript ends of hYAP by 5′ and 3′ RACE. (C) Amplification of full‐length hYAP coding sequence using nested PCR. (D) Structures of hYAP isoforms with the location of extra amino acids marked. The length of the open reading frames and the predicted molecular weights (MW) of the proteins are shown. * indicates the predicted isoforms. (E) The Pie chart presents the percentage of hYAP‐a (left) and hYAP‐b (right) splicing isoforms obtained from sequence analysis results of 127 and 102 clones, respectively. (F) The mRNA levels of hYAP‐a/hYAP‐b isoforms were detected by quantitative real‐time PCR. Data represent the mean ± SD; *n* = 3. ***P < 0.001. *P*‐values were calculated using Student's *t*‐test.

The AS events are usually assessed via RACE, PCR amplification of the full‐length cDNA, and sequencing. As the length of *hYAP* mRNA is about 5300 bp, RACE alone cannot ensure its complete amplification. In this study, we first amplified the 3′ end and 5′ end of *hYAP* using 3′‐RACE and 5′‐RACE, respectively, with specific primers (Fig. 1A). The result of RACE on a cDNA obtained from HEK293 cells is shown in Fig. 1B. The sequencing of the RACE products revealed the presence of two different TSSs at the 5′ end, each encoding a different exon 1, while no AS was observed at the 3′ end (exon 9). Based on these results, nested PCR primers were designed to amplify the full‐length coding sequence of *hYAP* (Fig. 1C). Through this approach, 23 *hYAP* isoforms were obtained and identified via sequencing. According to the differences in the first exon, the isoforms were divided into two groups (*hYAP‐a* and *hYAP‐b*). Each member was named according to the length of their coding amino acids, including newly identified five *hYAP‐a* isoforms (*hYAP‐a510*, *hYAP‐a506*, *hYAP‐a496*, *hYAP‐a494*, and *hYAP‐a490*) and nine *hYAP‐b* isoforms (*hYAP‐b330*, *hYAP‐b328*, *hYAP‐b316*, *hYAP‐b314*, *hYAP‐b312*, *hYAP‐b310*, *hYAP‐b288*, *hYAP‐b276*, and *hYAP‐b272*; Fig. 1D).

Unlike hYAP‐a, hYAP‐b isoforms lacked exon 1a, which encodes for the TEAD and the complete WW1 domain. However, both hYAP‐a and hYAP‐b include alternative 3′ splice site of exon 4 (which codes for amino acids GK), alternative 5′ splice site of exon 5 (which codes for amino acids DFFFLFIS), alternative 3′ splice site of exon 5 (which codes for amino acids VRPQ) and skipped exon of exon 4 or exon 6. Based on the conservation of AS events, we predicted the existence of *hYAP‐b332*, *hYAP‐b318*, and *hYAP‐b292* isoforms (Fig. 1D). In these splicing modes, the extension of exon 5 or preservation of exon 6 resulted in the disruption of leucine zipper motifs within the TAD, while the deletion of exon 4 resulted in the absence of the WW2 domain. Sequencing results from about 200 clones showed that *hYAP‐a504*/*b326* and *hYAP‐a488*/*b310* showed higher occurrence rates of 20%/24% and 20%/35%, respectively, followed by *hYAP‐a450*/*b272* (14%/11%). The occurrence rates for other *hYAP‐a*/*b* isoforms were lower than 10% (Fig. 1E). Quantitative real‐time PCR results showed that the total expression levels of all *hYAP‐a* isoforms were about 30 times higher than those of *hYAP‐b* isoforms (Fig. 1F).

### Analysis of expression patterns of hYAP isoforms

**Fig. 2:**
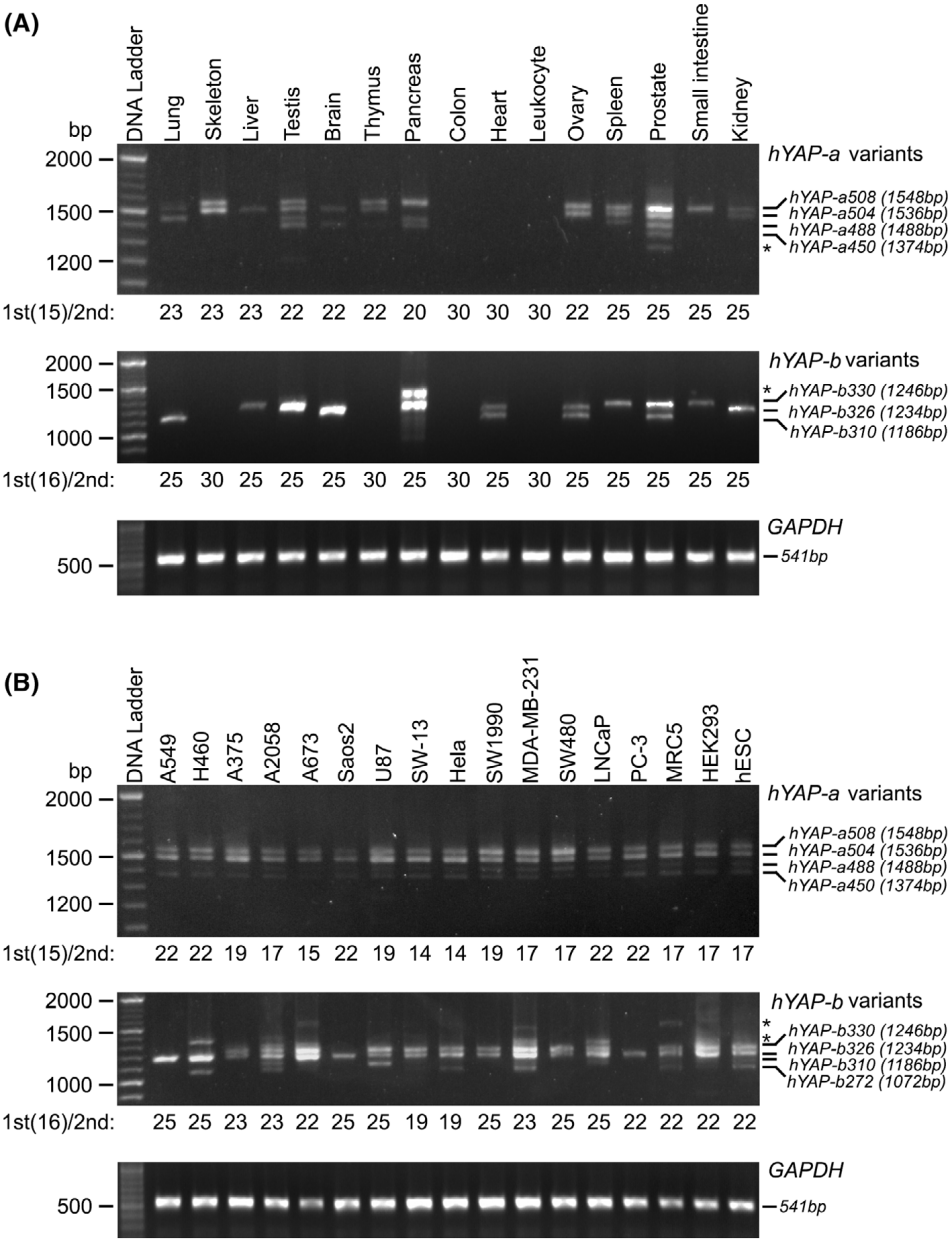
Expression of hYAP isoforms in different tissues and cell lines. (A) Expression pattern of *hYAP* isoforms in adult human tissues. cDNA panel of adult human tissue (lung, skeleton, liver, testis, brain, thymus, pancreas, colon, heart, leukocyte, ovary, spleen, prostate, small intestine, and kidney) were purchased from Clontech Laboratories, Inc. (B) Expression pattern of hYAP isoforms in different cell lines. RNAs were extracted from A549, H460, A375, A2058, A673, Saos2, U87, SW‐13, Hela, SW1990, MDA‐MB‐231, SW480, LNCaP, PC‐3, MRC5, HEK293, and hESCs, and then reverse transcribed to cDNA. hYAP‐a/b splicing isoforms were amplified for 15/16 cycles using the first set of primers (1st). The number of amplification cycles corresponding to the second set of primers is shown below the samples. * indicates the non‐specific amplification.

To analyze the expression pattern of hYAP isoforms, we performed nested PCR amplification of the cDNA obtained from various human tissues and cell lines, followed by gel electrophoresis. Both hYAP‐a and hYAP‐b were detected in the lung, liver, testis, brain, pancreas, ovary, spleen, prostate, small intestine, and kidney tissues, but with expression profiles of multiple isoforms. For instance, we can detect hYAP‐a504, hYAP‐a488, and hYAP‐b310 in the lung, while hYAP‐a504 and hYAP‐b326 in the liver. In addition, only hYAP‐a isoforms (hYAP‐a504 and hYAP‐a488) were detected in skeleton and thymus, and only hYAP‐b isoforms (hYAP‐b330 and hYAP‐b326) were detected in the heart. Neither hYAP‐a nor hYAP‐b isoforms were detected in colon and leukocyte samples (Fig. 2A). hYAP‐a isoforms were detected in all tested cell lines, exhibiting expression patterns similar to that in HEK293 cells. However, the expression pattern of hYAP‐b isoforms varied widely across different cell lines. For example, both hYAP‐b330 and hYAP‐b326 were detected in A375 (melanoma cell line), while only hYAP‐b326 was detected in A549 (lung cell line). The subtypes detected in U87 (glioma cell line) may include hYAP‐b330, hYAP‐b326, and hYAP‐b310 (Fig. 2B). These results indicated that the AS events comprehensively existed in normal tissues and cell lines; however, the splicing products were distinctly different.

### Subcellular localization of hYAP isoforms

**Fig. 3:**
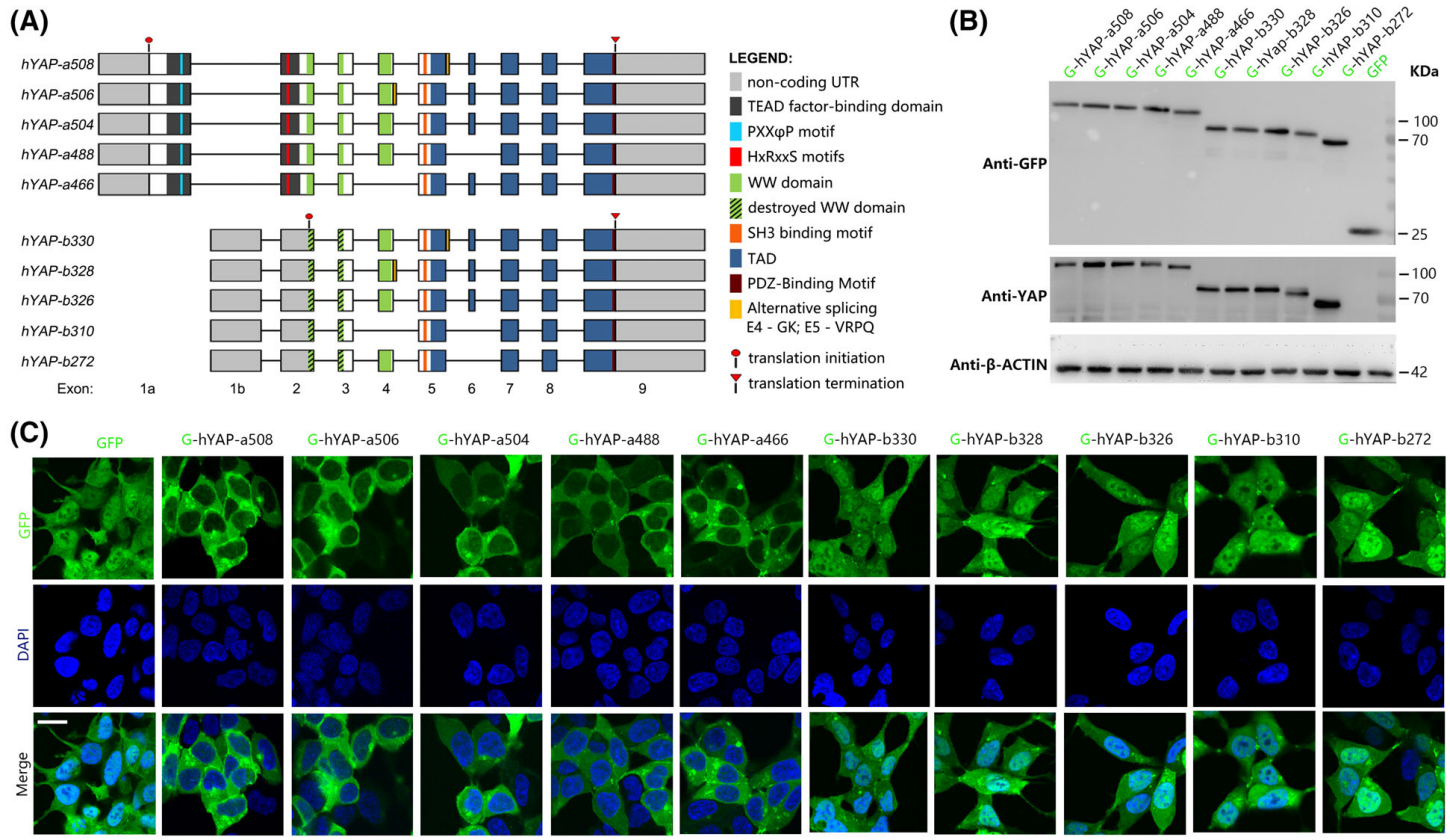
Recombinant expression and subcellular localization of hYAP isoforms. (A) Schematic diagram of 10 hYAP isoforms. Key structural features are indicated as WW domain (green), TAD (blue), TEAD‐binding domain (black), and PDZ‐binding pattern (dull red). (B) WB analysis of YAP and GFP proteins in the indicated cell lines. β‐ACTIN was used as an internal control. (C) Distinct subcellular localization of hYAP isoforms (green) in HEK293 cells. Cells were cultured in 5% FBS‐containing medium for 48 h, and the nuclei were counterstained with DAPI (blue). Scale bar: 20 μm.

The function of YAP is closely related to its subcellular localization. To assess whether the sequence of isoforms affected their subcellular localization, 10 isoforms with representative splicing modes and high expression levels were chosen for further analysis (Fig. 3A). Each GFP‐tagged YAP isoform was stably expressed alone in HEK293 cell lines. The expression of the fusion proteins was confirmed using WB with anti‐GFP or anti‐YAP antibody (Fig. 3B). Confocal images showed that hYAP‐a isoforms were mainly localized in the cytoplasmic region and sparsely in the nucleus, while hYAP‐b isoforms were equally distributed in both cytoplasm and nucleus (Fig. 3C). No significant difference was observed in subcellular localization among the hYAP‐a isoform overexpression (OE) groups and hYAP‐b isoform OE groups. These results suggested that amino acids 1–178 at the N‐terminal of the hYAP‐a isoforms, which are truncated in the hYAP‐b isoforms, might play a major role in the cytoplasmic retention of hYAP. In addition, our findings indicated that the integrity of the leucine zipper domain and WW2 domain might play no role in the subcellular localization of hYAP.

### Transcriptional activation ability of hYAP isoforms

**Fig. 4:**
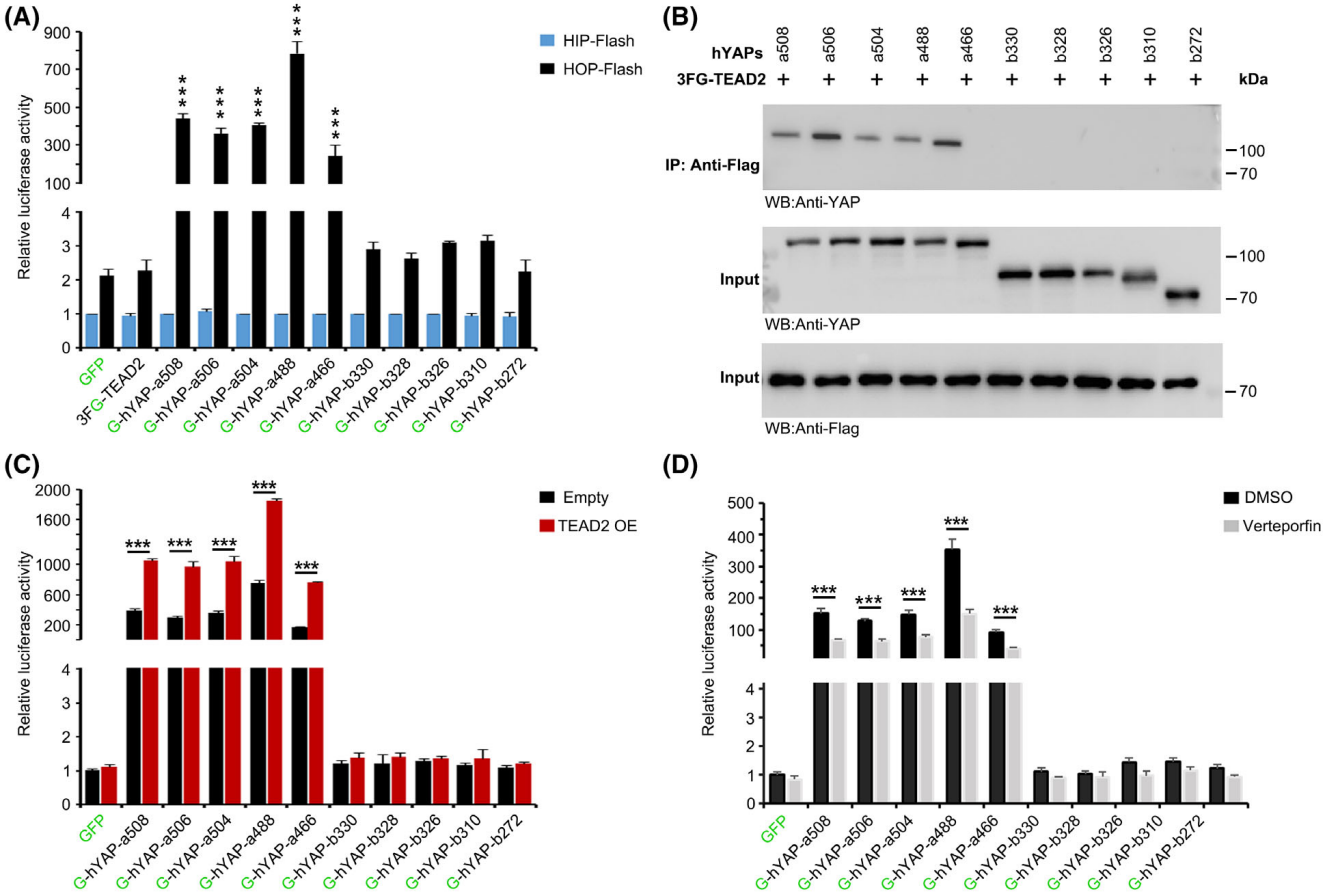
Transcriptional activation ability of hYAP isoforms. (A) The transcriptional activation abilities of different hYAP isoforms. Cells were co‐transfected with hYAP isoforms expression plasmids and HIP‐flash or HOP‐flash reporters. The transfected cells were cultured for 48 h, and then, the luciferase activity was measured. Data represent the mean ± SD; *n* = 3. ***P < 0.001. All *P*‐values were calculated using Dunnett's *t*‐test. (B) Interaction of TEAD2 with hYAP isoforms. The cells were co‐transfected with 3FG‐TEAD2 and GFP‐tagged hYAP isoform expression plasmids. Total cell lysates (input) and 3FG‐TEAD2 immunoprecipitation were blotted with the indicated antibodies. (C) TEAD2 OE promotes hYAP transactivation. HOP‐flash reporter relative luciferase activity was measured in the indicated cells transfected with either empty or TEAD2 OE vectors. (D) Disrupting the YAP‐TEAD interaction inhibits YAP transactivation. HOP‐flash (8 × WT TEAD‐binding sites) reporter relative luciferase activity was measured in the indicated cells either or not treated with verteporfin. Values were normalized to a Renilla luciferase control. The mean value of cells transfected with the GFP construct was set at 1.0. Data in C and D represent the mean ± SD; *n* = 3. ***P < 0.001. All *P*‐values were calculated using Student's *t*‐test.

**Fig. 5:**
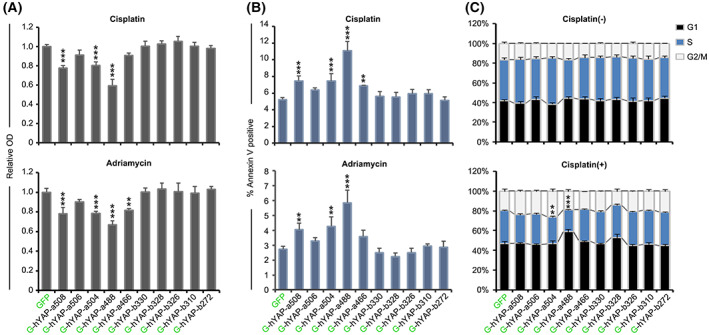
hYAP isoforms reveal additive proapoptotic effects with chemotherapeutics. (A) Pro‐cytotoxic effects of hYAP isoforms. Cells were cultured in a 5% FBS‐containing medium supplemented with either cisplatin or adriamycin for 72 h. Then, the cells were subjected to a CCK‐8 assay. (B) Effect of hYAP isoforms on apoptosis. Cells were cultured in a 5% FBS‐containing medium supplemented with either cisplatin or adriamycin for 72 h. Then, the cells were stained with annexin V‐APC and propidium iodide (PI) and analyzed by flow cytometry. (C) Effect of hYAP isoforms on cell cycle. Cells were cultured in a 5% FBS‐containing medium either or not supplemented with cisplatin for 72 h. Then, the cells were fixed, stained with PI, and analyzed by flow cytometry. Data in A–C represent the mean ± SD; *n* = 3. **P < 0.01; ***P < 0.001. All *P*‐values were calculated using Dunnett's *t*‐test.

As a cofactor, YAP cannot bind DNA directly [12]. Predominantly, YAP interacts with various DNA‐binding factors, such as members of the TEAD family (TEAD1–4), to regulate the transcription of target genes [14]. To determine the transcriptional activation ability of individual hYAP isoforms, we performed luciferase assay using a TEAD‐responsive HIP/HOP‐flash reporter system. The HOP‐flash carries eight copies of wild‐type TEAD‐binding sites linked to the minimal promoter and a luciferase reporter gene. The only difference in HIP‐flash is that it carries mutated TEAD‐binding sites [29]. Our results revealed that the luciferase activity of hYAP‐b isoforms was not significantly different from that of the control cells, whereas all hYAP‐a isoforms could activate HOP‐flash. Among them, hYAP‐a488 exhibited the strongest activation ability, while hYAP‐a466 exhibited the weakest. Furthermore, hYAP‐a508, hYAP‐a504, and hYAP‐a506 exhibited a decreasing trend of activation ability, although the difference between them was not significant (Fig. 4A). To determine whether hYAP isoforms interact differently with TEAD2, an IP assay was performed with HEK293 cells transiently co‐transfected with 3FLAG‐GFP‐tagged TEAD2 (3FG‐TEAD2) and GFP‐tagged hYAP isoforms. WB analysis indicated that hYAP‐b isoforms could not interact with TEAD2, whereas all hYAP‐a isoforms co‐precipitated with TEAD2 (Fig. 4B). However, there was no correlation between the binding affinities of hYAP‐a isoforms and their activation abilities. To exclude the possibility of varying endogenic TEAD2 expressions affecting variant enhancements of reporter activity exhibited by hYAP‐a isoforms, we repeated the luciferase assay with TEAD2 OE and verteporfin (a specific TEAD‐YAP interaction inhibitor)‐treated cells. Our results showed that the reporter activities were further increased by TEAD2 OE and reduced after treatment with verteporfin (Fig. 4C,D). Still, the hYAP‐a isoforms exhibited a consistent trend of enhancement, as shown in Fig. 5A. Taken together, these results indicated that all hYAP‐a isoforms could activate TEAD‐mediated transcription but with different co‐transcriptional regulatory abilities.

### Effects of hYAP isoforms on cell proliferation and chemosensitivity

Yes‐associated protein is an important regulator of cell proliferation, but the functional differences among different isoforms remain unknown. Our results showed that hYAP‐a508 OE enhanced the proliferation of HEK293 cells; however, hYAP‐a488 OE was detrimental to cell expansion. The proliferation ability of cells containing hYAP‐a506, hYAP‐a504, hYAP‐a466, and all hYAP‐b OE did not differ significantly from that of control cells (Fig. S1A). Cell cycle analysis revealed that the proportion of cells in the S phase was slightly reduced in the hYAP‐a488 OE cells. Other hYAP isoforms did not significantly affect the cell cycle distribution (Fig. 5C).

Chemotherapeutic agents, such as cisplatin and adriamycin, can lead to cell apoptosis by inducing DNA damage. Previous studies have shown that YAP OE enhanced the sensitivity of cells to these agents [20]. To investigate whether hYAP isoforms exhibited varying effects on cell chemosensitivity, the viability, and apoptosis of hYAP isoform OE cells were assessed using CCK‐8 assay and annexin V‐APC staining with cisplatin or adriamycin treatment. The results revealed no significant difference between the hYAP‐b OE cells and the control cells. However, hYAP‐a508, hYAP‐a504, and hYAP‐a488 OE exhibited significantly enhanced pro‐cytotoxic effects on cells. Among them, the strongest cytotoxicity was induced by hYAP‐a488, followed by hYAP‐a508 and hYAP‐a504. hYAP‐a506 and hYAP‐a466 also showed slightly enhanced pro‐cytotoxic effects on cells, although the enhancements were not statistically significant (Fig. 5A,B). These results suggested that the 178 amino acids truncated at the N‐terminal of hYAP‐b isoforms might contain functional domains critical to the pro‐cytotoxic effect of YAP, further enhanced by the intact leucine zipper. Moreover, the results of the analysis of cell cycle distribution and BAX expression levels were consistent with those of the cytotoxicity assays (Fig. 5C and Fig. S1B,C). These results excluded the possibility of any systemic error in our analytical methods.

### Effect of hYAP isoforms on transcriptional activation ability of P73

**Fig. 6:**
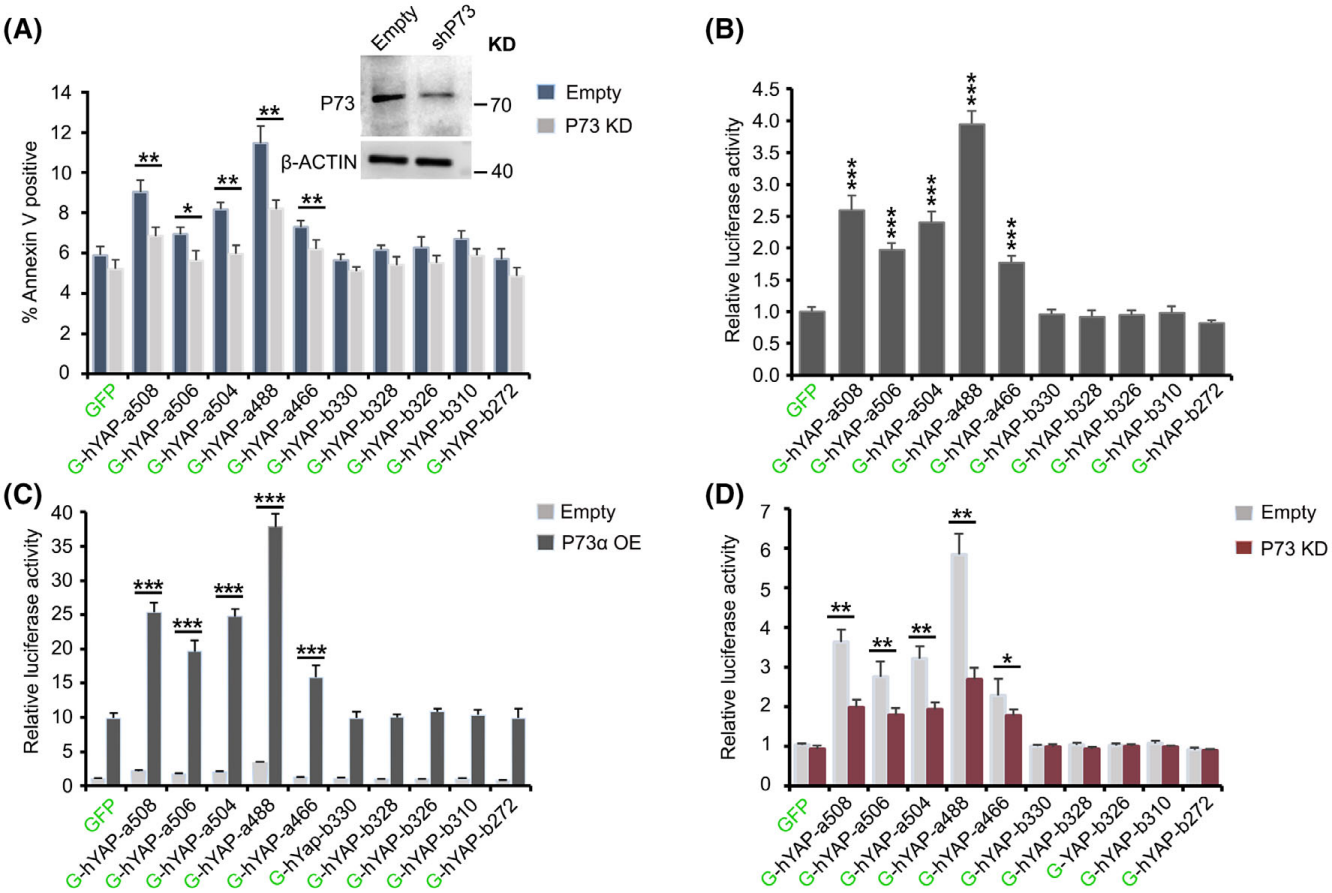
hYAP isoforms induce the proapoptotic effects via the P73‐mediated pathway. (A) P73 KD attenuates hYAP‐induced proapoptotic effects. Cells cultured in a 5% FBS‐containing medium supplemented with cisplatin for 72 h were stained with annexin V‐APC and analyzed by flow cytometry. The efficiency of P73 KD was assessed using WB analysis with the indicated antibodies. (B) hYAP isoforms exhibit varying effects on the transcriptional activation ability of P73. The cells were co‐transfected with hYAP isoforms and P73 luciferase reporter plasmids. After incubation for 48 h, cell extracts were prepared and subjected to luciferase activity determination. Data represent the mean ± SD; *n* = 3. ***P < 0.001. All *P*‐values were calculated using Dunnett's *t*‐test. (C) hYAP transactivation was enhanced by P73α OE. P73 reporter relative luciferase activity was measured in the indicated cells transfected with either empty or 73α OE vectors. (D) hYAP transactivation was attenuated by P73 KD. P73 reporter relative luciferase activity was measured in the indicated cells transfected with either Luc KD or P73 KD vectors. Values were normalized to a Renilla luciferase control. The mean value of cells transfected with the GFP construct was set at 1.0. Data in A, C, and D represent the mean ± SD; *n* = 3. *P < 0.05; **P < 0.01; ***P < 0.001. All *P*‐values were calculated using Student's *t*‐test.

YAP‐induced chemo‐sensitization probably involves several different mechanisms [20, 30, 31]. Previous reports have shown that overexpression of YAP might increase the transcriptional activation ability of P73, which, in turn, promotes the expression of target apoptotic genes (such as *BAX and PUMA*) [32, 33, 34, 35]. To ascertain whether the pro‐cytotoxic effect induced by all hYAP isoforms is mediated via P73, we created RNAi‐mediated P73 knockdown (KD) cell lines for all isoforms. Annexin V‐APC staining assay showed that P73 KD rescued the cells from apoptosis induced by all hYAP‐a isoforms under cisplatin treatment (Fig. 6A). This result indicated that the effects of hYAP‐a isoforms on the chemosensitivity of the cells to cisplatin were primarily mediated via P73.

To further explore the effect of YAP isoforms on P73‐mediated transcriptional activation ability, we constructed a P73‐responsive luciferase reporter system carrying a *BAX* gene promoter [28]. The luciferase assay revealed that all hYAP‐a isoforms significantly activated the reporter system. Among them, hYAP‐a488 exhibited the most significant enhancement of luciferase activity, followed by hYAP‐a508, hYAP‐a504, hYAP‐a506, and hYAP‐a466 (Fig. 6B). The elevated reporter activity of hYAP‐a isoforms was attenuated by P73 KD, while P73α OE further enhanced the reporter activity (Fig. 6C,D). These results suggested that different hYAP‐a isoforms might induce varying P73 transactivation abilities, which, in turn, leads to cells developing varying drug sensitivities.

## Discussion

AS is an important post‐transcriptional regulation mechanism that enables one gene to produce multiple isoforms, thus increasing the complexity of the transcriptome and proteome [36, 37]. As an important effector of the Hippo pathway, YAP plays a significant role in organogenesis, embryonic development, and tumorigenesis [9, 38, 39]. Identification of YAP isoforms and elucidation of their functions are key prerequisites for analyzing their roles in determining cell fate.

Previous studies have confirmed the existence of nine isoforms of hYAP [23, 40]. In this study, we identified 23 hYAP isoforms in HEK293 cells. To the best of our knowledge, 14 of these isoforms were reported for the first time. Sequence analysis of hYAP isoforms revealed that in addition to the three reported splicing types, including the alternative 3′ splice site of exon 5 (which encodes for amino acids VRPQ), skipped exon 4, and retained exon 6 [23], we also identified two novel splicing modes at the alternative 5′ splice site of exon 5 (which encodes for amino acids DFFFLFIS) and the alternative 3′ splice site of exon 4 (which encodes for amino acids GK). Moreover, we found that several YAP‐related AS events were conserved in humans and mice, such as the extension of exon 5 and preservation of exon 6 [21]. Accordingly, we speculated the existence of hYAP‐b332, hYAP‐b318, and hYAP‐b292. However, whether these hYAP isoforms actually exist still needs to be confirmed in further studies.

Yes‐associated protein activity is primarily regulated by its subcellular localization. Extensive research has shown that when the Hippo signaling pathway is activated, YAP is phosphorylated at serine 127, the phosphorylated YAP is then captured by 14–3–3 protein and retained in the cytosol. When the Hippo pathway is inhibited, this YAP site is dephosphorylated and separated from 14–3–3 protein, and the YAP protein is then translocated to the nucleus [19, 39, 41]. However, YAP lacks a nuclear localization signal, and its nucleus translocation is dependent on binding to other proteins. It has been reported that ZO‐2 (a PDZ domain‐containing protein) and MAML1/2 (including a conserved PPxY‐interacting motif) can interact with the PDZ‐binding motif or WW domain of YAP to facilitate YAP translocation into the nucleus [42, 43, 44]. Nonetheless, whether the changes in amino acid sequences of different hYAP isoforms affect their subcellular localization remains uncertain. In this study, all five hYAP‐a isoforms were found to be primarily localized in the cytoplasm when the cell confluence reached nearly 80% fusing (Hippo pathway is activated), while the five hYAP‐b isoforms were evenly distributed in cytoplasm and nucleus. Given that the serine 127 phosphorylation site was unique to hYAP‐a, these results indicated that this serine residue might play a key role in the cytoplasmic retention of hYAP. Moreover, no intragroup differences were observed in subcellular localizations of hYAP‐a isoforms and hYAP‐b isoforms. Therefore, hYAP localization might not be affected by the alternative 3′ splice site of exon 5 (with the addition of amino acids VRPQ), alternative 3′ splice site of exon 4 (with the addition of amino acids GK), the deletion of the WW2 domains, and the integrity of the leucine zipper.

The luciferase assay showed that all hYAP‐a isoforms activated TEAD‐mediated transcription but to varying degrees. Among them, hYAP‐a488 exhibited the strongest transactivation ability, while hYAP‐a466 exhibited the weakest. Protein structural analysis showed that only hYAP‐a488 had a complete leucine zipper and two WW domains. The other hYAP‐a isoforms contained disrupted leucine zippers, while hYAP‐a466 also lacked the WW2 domain. These results suggested that the leucine zipper and the WW2 domain contributed to the transactivation ability of hYAP. Thus, we speculated that hYAP‐a496, hYAP‐a494, hYAP‐a492, and hYAP‐a490, all of which carried the complete leucine zipper and two WW structural domains, may also exhibit stronger transactivation ability than the hYAP isoforms that lack these structures. In addition, the sequentially decreasing regulatory ability was observed for hYAP‐a508, hYAP‐a504, and hYAP‐a506, suggesting that the transactivation ability of hYAP enhanced after the alternative 3′ splice site of exon 5 (with the addition of amino acids VRPQ), but repressed by alternative 3′ splice site of exon 4 (with the addition of amino acids GK).

Previous research has shown that YAP plays a crucial role in cellular sensitivity to chemotherapeutic agents. On the one hand, YAP can promote cell growth and proliferation by interacting with TEADs or AP‐1 or by decreasing AKT‐mediated nuclear localization of P27Kip1 [45, 46, 47]. On the other hand, YAP can also induce apoptosis by interacting with P73 and upregulating the expression of P73 downstream targets (such as *P21*, *BAX*, and *PUMA*) [20, 35, 48]. The cellular behavior or phenotype is mediated by a balance of these effects. Currently, it is unclear whether YAP isoforms exerted varying effects on cell proliferation and drug resistance. In this study, we observed that hYAP‐a overexpression exhibited little effect on cell proliferation, which might be attributed to the inability of the overexpressed hYAP‐a isoforms to translocate into nuclei under conventional culture conditions (Fig. 3C). Nevertheless, our results clearly showed that hYAP‐a508 can promote the proliferation of HEK293 cells, while hYAP‐a488 exerted inhibitory effects, suggesting each hYAP isoform might act through distinct molecular mechanisms. In the presence of cisplatin or adriamycin, hYAP‐a isoforms significantly increased the transactivation ability of P73 and enhanced cellular sensitivity to chemotherapeutic drugs. This finding was consistent with the results of previous studies [32, 35]. Interestingly, although hYAP interacts with TEAD and P73 via different domains, hYAP‐a isoforms showed similar transactivation effects on these two transcriptional factors. The most prominent transactivation was exhibited by hYAP‐a488, followed by hYAP‐a508, hYAP‐a504, hYAP‐a506, and hYAP‐a466. These results suggested that the co‐transcriptional regulatory effect of each isoform was conserved.

hYAP‐b isoforms were widely distributed in normal human tissues and tumor cell lines. However, none of the hYAP‐b isoforms in this study were found to exhibit any significant biological effects. Previous studies have found that WW1 might be the main domain that mediates the interaction of YAP with other proteins [16]. The hYAP‐b isoforms lacked both TEAD and WW1 domains. Therefore, the lack of any effects of hYAP‐b isoforms on cell chemosensitivity might be attributed to their inability to interact with P73. However, it has also been demonstrated that YAP can interact with other proteins through the SH3 domain, PDZ‐binding motif, etc. [44, 49]. Moreover, hYAP‐b isoforms also carry a complete C‐terminal transactivation functional domain. So, it can be speculated that hYAP‐b isoforms might be involved in other biological processes. The functional characterization and related mechanisms of the hYAP‐b need to be further studied.

Taken together, we identified 23 hYAP isoforms in HEK293 cells and found that the different molecular structures resulting from variant splicing modes might affect their subcellular localizations, transactivation abilities, and cellular drug resistance‐imparting abilities. These findings will not only lay the foundation for understanding the function and regulatory mechanisms of YAP but also help elucidate the role of the Hippo signaling pathway in several diseases.

## Conflict of interest

The authors declare no conflict of interest.

### Peer review

The peer review history for this article is available at https://www.webofscience.com/api/gateway/wos/peer‐review/10.1002/2211‐5463.13618.

## Author contributions

LLL and JLZ designed and carried out the experiments, analysis, and interpretation of the data and manuscript writing. JLW, JQW, YPT, YXX, YDC, and MY provided technical assistance. YY, RY, XYW, JJW, WW, CZ, and YH analyzed and interpreted the data. RJ, LX, and YR designed the experiments and directed the study. All authors have read and approved the final version of the manuscript.

## Supporting information


**Fig. S1.** Effects of hYAP isoforms on cell proliferation and apoptosis. (A) Effects of hYAP isoforms on cell proliferation were analyzed by CCK‐8 assay. (B‐C) The mRNA and protein expression levels of BAX in the indicate cell lines after cisplatin (B) and adriamycin (C) treatment. Data in A, B, and C were represented as mean ± SD; *n* = 3. *P < 0.05; **P < 0.01; ***P < 0.001. All P‐values were calculated using Dunnett's t‐test.
**Table S1.** Primers for plasmid construction.
**Table S2.** Primers for semi‐quantitative PCR analysis.
**Table S3.** Primers for quantitative real‐time PCR analysis.
**Table S4.** Antibody details.

## Data Availability

Strains and plasmids are available upon request. Sequence data are available at GenBank and the accession numbers listed: hYAP‐a510‐OL825682, hYAP‐a508‐OL825683, hYAP‐a506‐OL825684, hYAP‐a504‐OL825685, hYAP‐a496‐OL825686, hYAP‐a494‐OL825687, hYAP‐a492‐OL825688, hYAP‐a490‐OL825689, hYAP‐a488‐OL825690, hYAP‐a470‐OL825691, hYAP‐a466‐OL825692, hYAP‐a454‐OL825693, hYAP‐a450‐OL825694, hYAP‐b330‐OL825695, hYAP‐b328‐OL825696, hYAP‐b326‐OL825697, hYAP‐b316‐OL825698, hYAP‐b314‐OL825699, hYAP‐b312‐OL825700, hYAP‐b310‐OL825701, hYAP‐b288‐OL825702, hYAP‐b276‐OL825703, and hYAP‐b272‐OL825704.
