# Supplementary material for: Identification and functional comparison of novel alternatively spliced isoforms of human YAP
Source: FEBS Open Bio. 2023 May 8;13(6):1001–14. doi: 10.1002/2211-5463.13618 (PMC10240341; doi:10.1002/2211-5463.13618)
Supplement: Supplementary file 1 — Fig. S1. Effects of hYAP isoforms on cell proliferation and apoptosis. (A) Effects of hYAP isoforms on cell proliferation were analyzed by CCK‐8 assay. (B‐C) The mRNA and protein expression levels of BAX in the indicate cell lines after cisplatin (B) and adriamycin (C) treatment. Data in A, B, and C were represented as mean ± SD; n = 3. *P < 0.05; **P < 0.01; ***P < 0.001. All P‐values were calculated using Dunnett's t‐test. Table S1. Primers for plasmid construction. Table S2. Primers for semi‐quantitative PCR analysis. Table S3. Primers for quantitative real‐time PCR analysis. Table S4. Antibody details. [file FEB4-13-1001-s001.pdf]

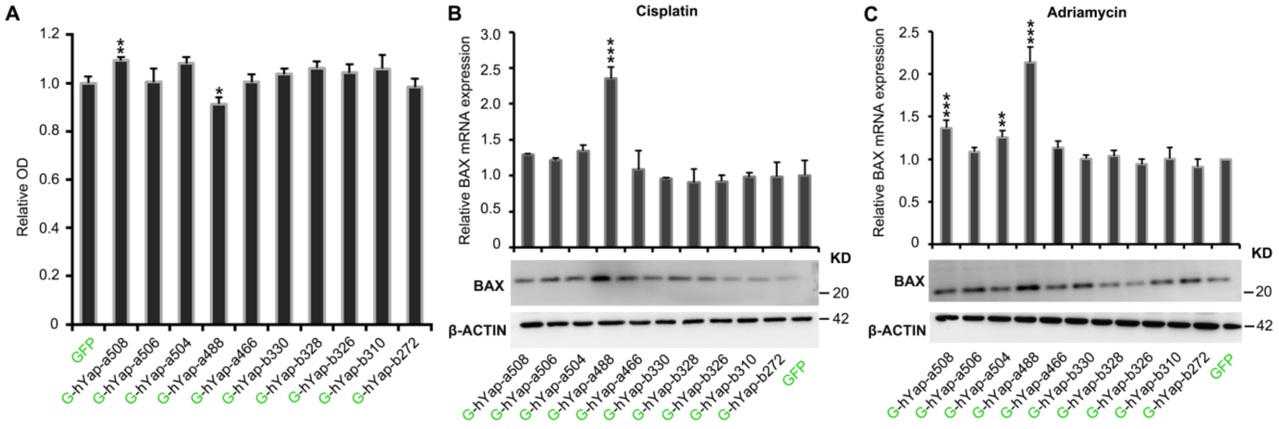

Figure S1. Effects of hYAP isoforms on cell proliferation and apoptosis.

(A) Effects of hYAP isoforms on cell proliferation were analyzed by CCK-8 assay.

(B-C) The mRNA and protein expression levels of BAX in the indicate cell lines after cisplatin (B) and adriamycin (C) treatment. Data in A, B, and C were represented as mean ± SD; n = 3. *$P < 0.05$; **$P < 0.01$; ***$P < 0.001$. All *P*-values were calculated using Dunnett's t-test.

Table S1. Primers for plasmid construction

Name	Primer Sequence (5' - 3')	
	Forward	Reverse
pPyCAGIH-GFP-hYAP	ACTCGAGCTAGCGGAGGAGGAGGAATGGATCCCGGGCAG	AGCGGCCGCCTATAACCATGTAAG
pPyIZ-3FLAG-hP73α	AGCTAGCGCCCAGTCCACCGCCACCTC	TGCTAGCTCAGTGGATCTCGGCCTCCG
pPyCAGIZ-3FLAG-GFP-TEAD2	AGCTAGCATGGGGGATCCCCGGACTG	AGCTAGCTCAGTCCCTGACCAGGCGGTAG
pGL3-Enhancer-hBAX	TTGAACGCGTGCGAGGATCGCTTGAGTCTG	TTCCAAGCTTCTAATGCCTTCATTTATCCAG

Table S2. Primers for semi-quantitative PCR analysis

Gene	Primer Sequence (5' - 3')	
	Forward	Reverse
GAPDH	AGGTCGGTGTGAACGGATTTG	TGTAGACCATGTAGTTGAGGTCA
hYAP-a (outer)	AGTTGGAGGGAAAAAGTTCTCAG	TGTCTCCTTAGATCCTTCACAG
hYAP-a (inner)	GGAGGCAGAAGCCATGGATC	TGAGGGCTCTATAACCATGTAAG
hYAP-b (outer)	ACTGCAATGTAGTTAGCCCACTCG	TGTCTCCTTAGATCCTTCACAG
hYAP-b (inner)	ATTTTGAGTCCCACCATCCTGC	TGAGGGCTCTATAACCATGTAAG

Table S3. Primers for quantitative real-time PCR analysis

Gene	Primer Sequence (5' - 3')	
	Forward	Reverse
GAPDH	AGGTCGGTGTGAACGGATTTG	TGTAGACCATGTAGTTGAGGTCA
hYAP-a	GGAGGCAGAAGCCATGGATC	AGAGAAGCTGGAGAGGAATGAG
hYAP-b	GGAGGCAGAAGCCATGGATC	AGAGAAGCTGGAGAGGAATGAG
BAX	GTTGTCGCCCTTTTCTACTTTG	CCATGATGGTTCTGATCAGTTC

Table S4. Antibody details

	**Antibody**	**Source**	**Cat. no.**	**Dilution**
**Primary**	YAP	R&D	MAB8094	1:1000(WB)
	BAX	Cell SignalingTechnology	2772s	1:1000(WB)
	P73	Santa Cruz	Sc-56190	1:1000(WB)
	β-ACTIN	Sigma	AF3369	1:10000 (WB)
	GFP	Beyotime	AF0157	1:1000(WB)
	FLAG	Beyotime	AF519	1:200 (IP);<br>1:1000 (WB)
**Secondary**	Goat anti-mouse IgG<br>Goat anti-rabbit IgG	Beyotime	A0216<br>A0208	1:2000 (WB)
